# Caloric restriction induces energy-sparing alterations in skeletal muscle contraction, fiber composition and local thyroid hormone metabolism that persist during catch-up fat upon refeeding

**DOI:** 10.3389/fphys.2015.00254

**Published:** 2015-09-16

**Authors:** Paula B. M. De Andrade, Laurence A. Neff, Miriam K. Strosova, Denis Arsenijevic, Ophélie Patthey-Vuadens, Leonardo Scapozza, Jean-Pierre Montani, Urs T. Ruegg, Abdul G. Dulloo, Olivier M. Dorchies

**Affiliations:** ^1^Department of Medicine, Physiology, University of FribourgFribourg, Switzerland; ^2^Pharmaceutical Biochemistry, Geneva-Lausanne School of Pharmaceutical Sciences, University of Geneva, University of LausanneGeneva, Switzerland; ^3^Pharmacology, Geneva-Lausanne School of Pharmaceutical Sciences, University of Geneva, University of LausanneGeneva, Switzerland

**Keywords:** thermogenesis, catch-up growth, weight regain, obesity, deiodinase, skeletal muscle, contraction-relaxation, rat

## Abstract

Weight regain after caloric restriction results in accelerated fat storage in adipose tissue. This catch-up fat phenomenon is postulated to result partly from suppressed skeletal muscle thermogenesis, but the underlying mechanisms are elusive. We investigated whether the reduced rate of skeletal muscle contraction-relaxation cycle that occurs after caloric restriction persists during weight recovery and could contribute to catch-up fat. Using a rat model of semistarvation-refeeding, in which fat recovery is driven by suppressed thermogenesis, we show that contraction and relaxation of leg muscles are slower after both semistarvation and refeeding. These effects are associated with (i) higher expression of muscle deiodinase type 3 (DIO3), which inactivates tri-iodothyronine (T_3_), and lower expression of T_3_-activating enzyme, deiodinase type 2 (DIO2), (ii) slower net formation of T_3_ from its T_4_ precursor in muscles, and (iii) accumulation of slow fibers at the expense of fast fibers. These semistarvation-induced changes persisted during recovery and correlated with impaired expression of transcription factors involved in slow-twitch muscle development. We conclude that diminished muscle thermogenesis following caloric restriction results from reduced muscle T_3_ levels, alteration in muscle-specific transcription factors, and fast-to-slow fiber shift causing slower contractility. These energy-sparing effects persist during weight recovery and contribute to catch-up fat.

## Introduction

Mammals adapt to food scarcity by increasing the efficiency of energy utilization. This has been documented in longitudinal studies of starvation and caloric restriction in humans as well as animals (Keys et al., 1950; Keesey and Powley, 2008) and is regarded as an outcome of adaptive processes to a deficit in energy intake. There is evidence that this ability persists, at least in part, during weight recovery upon refeeding (Boyle et al., 1978; Harris and Martin, 1984; Hill et al., 1984) and that the energy thus conserved is directed at accelerating specifically the recovery of the body's adipose tissue rather than that of other tissues in animals (Dulloo and Girardier, 1990; MacLean et al., 2004; Evans et al., 2005) and in humans (Dulloo and Jacquet, 1998; Weyer et al., 2000). This preference for “catch-up fat” is viewed as a result of a feedback loop between adipose tissue and thermogenesis (Dulloo and Jacquet, 2001); it probably evolved to optimize survival capacity during an ancestral life characterized by periodic food shortage. Nowadays, it is the key factor causing higher body fat gain relative to lean tissue and is commonly observed in adults after malnutrition, weight loss cures, anorexia nervosa and cancer-cachexia (Dulloo and Jacquet, 2001). This preferential catch-up fat phenomenon has also been linked to the hyperinsulinemic state of catch-up growth and the associated risks for later development of the metabolic syndrome (Crescenzo et al., 2003; Dulloo et al., 2006).

While the underlying mechanisms of the thrifty metabolism and catch-up fat are still poorly understood, studies using a rat model of semistarvation-refeeding in which catch-up fat is driven solely by suppressed thermogenesis (Dulloo and Girardier, 1990; Crescenzo et al., 2003) have implicated a role of impaired skeletal muscle metabolism for the following reasons: First, *in vivo* assessment of insulin-stimulated tissue glucose utilization during weight recovery indicates that glucose uptake is increased in white adipose tissue but reduced in skeletal muscle, suggesting a preference for storage via *de novo* lipogenesis and, perhaps, muscular insulin resistance (Cettour-Rose et al., 2005). Second, assessments of the mitochondrial subpopulations and oxidative capacities in hindlimb muscle suggest that the subsarcolemmal mitochondrial mass and its oxidative capacity, known to be diminished during caloric restriction, remained lower after refeeding (Crescenzo et al., 2006). Third, the circulating level of the main active thyroid hormone, tri-iodothyronine (T_3_)—for which skeletal muscle is a major target—is diminished during caloric restriction and tends to remain low in refed animals during the energy-sparing phase of driving catch-up fat (Mainieri et al., 2006; Summermatter et al., 2008).

Given the evidence that this hypothyroid state is associated with diminished ATP turnover but increased mechanical efficiency (Lambert et al., 1951; Wiles et al., 1979; Johansson et al., 2003), we addressed the question of whether the slowed kinetics of skeletal muscle contraction-relaxation, known to occur during caloric restriction (Russell et al., 1983a,b, 1984a; Chan et al., 1986; Lewis et al., 1986; Pichard and Jeejeebhoy, 1988; Sieck et al., 1989; Nishio and Jeejeebhoy, 1991; Mijan de la Torre et al., 1993; Bissonnette et al., 1997), persist during weight recovery and might hence be linked to the thrifty metabolism driving catch-up fat.

Using our previously validated rat model of semistarvation-refeeding (Dulloo and Girardier, 1990; Crescenzo et al., 2003, 2006; Cettour-Rose et al., 2005; Mainieri et al., 2006; Summermatter et al., 2008), we found altered *in vivo* contractile properties of the hindlimb muscle not only at the end of a 2 week period of caloric restriction but also after 1 week of refeeding. These functional changes correlated with a fast-to-slow fiber transition, impaired expression of transcription factors involved in the development of slow-twitch muscles, namely calcineurin, peroxisome proliferator-activated receptor gamma coactivator 1-α (PGC1-α), and forkhead box protein O1 (FoxO1), and with a decreased rate of muscular T_3_ synthesis linked to changes in the levels of the deiodinases DIO1, DIO2, and DIO3. The reduced T_3_ synthesis, the slower rate of contraction-relaxation and the alterations in muscle size and shape persisted after refeeding.

## Materials and methods

### Animals and diet

A total of 40 male Sprague Dawley rats (Janvier Labs, le Genest-Saint-Isle, France) were used in this study. Rats were housed single in a temperature-controlled room (22 ± 1°C) with a 12-h light/12-h dark cycle. They were maintained on chow diet (Provimi-Kliba, Kaiseraugst, Switzerland) consisting, by energy, of 24% protein, 66% carbohydrates, and 10% fat, and had free access to water. Animals were maintained in accordance with the regulations and guidelines of the Department of Medicine (University of Fribourg) for the care and use of laboratory animals. All the experimental procedures involving animals were approved by ethical committees and veterinary offices of the States of Fribourg and Geneva.

### Design of the study

The experimental design was similar to that previously described in establishing a rat model for studying changes in energy expenditure that occur during accelerated fat recovery (i.e., catch-up fat) upon refeeding after growth arrest (Dulloo and Girardier, 1990; Crescenzo et al., 2003; Cettour-Rose et al., 2005; Mainieri et al., 2006). This approach allows investigating changes in thermogenesis specific for catch-up fat in the absence of confounding variables, such as food intake and differential rates of protein gain. In brief, 7-week-old rats were food-restricted at 50% of their spontaneous food intake for 2 weeks (semistarved rats, SS rats), after which they were refed (RF rats) the amount of chow corresponding to the spontaneous chow intake of control rats matched for weight at the onset of refeeding (control rats). Under these conditions, the refed animals showed a similar gain in lean mass, but an about 2-fold increase in body fat gain as compared to controls over a period of 2 weeks, due to 10–13% lower energy expenditure resulting from suppressed thermogenesis (Dulloo and Girardier, 1990; Crescenzo et al., 2003). The present study was conducted on SS rats and their fed controls (C_SS_) at the end of 2 weeks of semistarvation, as well as on RF rats and their weight-matched controls (C_RF_) after 1 week of refeeding.

### Isometric force recordings

Isometric force recordings of the triceps surae muscle were performed *in situ* as previously described in mice (Dorchies et al., 2006, 2013; Reutenauer et al., 2008; Hibaoui et al., 2011; Nakae et al., 2012; Reutenauer-Patte et al., 2012) but were adapted to rats: a larger platform for accommodating larger animals on the force equipment setup was constructed, and the stimulus controller was modified to deliver stimulations up to 80 V instead of 40 V. In brief, electrical stimulations were delivered to the triceps surae of deeply anesthetized rats. Time to peak (TTP), time for half-relaxation from the peak (RT1/2), absolute and specific phasic twitch peak tension (*Pt*) and tetanic tension (*Po*), force-frequency relationship, and fatigability of muscle exposed to repetitive tetanic contractions were determined. Experimental details are provided in Supplementary Material.

### Tissue sampling

Rats were euthanized immediately after the force parameters were recorded. The triceps surae muscle from the stimulated leg was carefully dissected and weighed for the calculation of total muscle cross-sectional area (CSA) (see Supplementary Material). The gastrocnemius muscle was chosen for further analyses because it accounts for about 80% of the triceps mass, and therefore, it is the muscle that contributes the most to the analyzed force parameters. Gastrocnemius muscles are made up of fast-twitch fibers located in the external, glycolytic regions, and slow-twitch fibers that define an oxidative area in the deep region of the muscle. Gastrocnemius muscles from stimulated legs were embedded in Tissue-Tek® OCT™ compound, frozen in liquid nitrogen, and stored at −80°C until processed for histology. Gastrocnemius muscles from non-stimulated legs were also dissected, frozen in liquid nitrogen, and stored at −80°C for biochemical and molecular assays.

### Histology and immunofluorescent labeling

#### Muscle sections

Serial transverse sections (10 μm thick) were prepared from the largest aspect of the gastrocnemius muscle belly (approximately one-third of muscle length from proximal end) using an HM 560 M cryostat (Microm, Volketswil, Switzerland) and collected on SuperFrost Plus slides. Every slide held sections of either SS or RF samples together with their respective controls. Slides were stored at −80°C until processed.

#### Hematoxylin-eosin staining

The muscle sections were stained with hematoxylin-eosin according to classical procedures (Dorchies et al., 2006, 2013; Nakae et al., 2012; Reutenauer-Patte et al., 2012) to assess overall muscle morphology. Activities of NADH-reductase, succinate dehydrogenase and cytochrome C oxidase were evaluated by enzyme histochemistry.

#### Immuno-labeling of myosin heavy chains

The muscle sections were exposed to antibodies specific to myosin heavy chain isoforms 1, 2A, and 2B, which are markers of muscle fibers of type I, IIA and IIB, respectively, essentially as described (Dorchies et al., 2013). Technical details about the immuno-labeling procedures are provided in Supplementary Material.

#### Pictures

For each gastrocnemius, high-resolution pictures (final magnification 50X) were taken from the very same area of the deep, oxidative region with an AxioCam MRc digital camera (Zeiss, Feldbach, Switzerland) mounted on an inverted microscope (Zeiss Axiovert 200 M) equipped for epifluorescence. Visualization of green (AF_488_), red (AF_594_), and blue (Hoechst) fluorescence was achieved with specific filter sets (Zeiss). Black and white pictures were pseudo-colored and overlayed with the MetaMorph software (Visitron Systems, Puchheim, Germany) (Dorchies et al., 2013). Pictures were coded so that the investigators were blind to the group details.

#### Analysis of MyHC-positive fibers

Fiber analysis was performed as described in detail elsewhere (Dorchies et al., 2013). The fibers labeled positively for MyHC 1-, 2A-, or 2B were assigned as fibers of type I, IIA, or IIB, respectively. The remaining fibers (negative for MyHC 1, 2A, and 2B) were designated as type IIX fibers. Fibers were counted using built-in tools of the ImageJ software (NIH, MD, USA) and expressed as the percentage of the total number of fibers (Dorchies et al., 2013). For each MyHC isoform, one picture (approximately 1900 fibers) taken from the deep area of each cross-section was analyzed. In order to minimize counting errors, the full analysis was performed by 2 investigators (PBMA and OPV) and the results were averaged.

### Net T_3_ neogenesis assay

The kinetics of thyroid hormone metabolism in skeletal muscle were assessed *in vitro* using the method of Kaplan and Utiger (Kaplan and Utiger, 1978) by incubating muscle homogenates in Tris buffer at 37°C. The T_3_ neogenesis reaction was started by adding T_4_ (1.3 μM) dissolved in PBS containing 0.25% BSA. Aliquots of the homogenate were removed after 0, 5, 10, 15, and 30 min, the reaction was stopped by adding 95% ethanol and samples were stored at 4°C until assayed for thyroid hormone content using a T_3_/T_4_ enzyme immunoassay kits (Diagnostic Systems Laboratories, Webster, TX, USA).

### Semi-quantitative determination of muscle proteins by western blot

The expression levels of gastrocnemius muscle proteins were determined by Western blots according to standard procedures described in detail elsewhere (Reutenauer-Patte et al., 2012; Dorchies et al., 2013). In brief, gastrocnemius muscles were pulverized in liquid nitrogen-cooled mortars and extracts were prepared in Guba-Straub buffer containing 1% Triton X-100 and 0.1% 2-mercaptoethanol. Protein content in the extracts was assayed and adjusted to 3 μg/μL with 2x reducing Laemmli buffer. Thirty μg of the extracts were resolved by SDS-PAGE before being transferred to nitrocellulose membranes. Equal loading and transfer efficiency were verified by staining with Ponceau Red. The membranes were then exposed to primary antibodies specific for selected muscle proteins, to appropriate HRP-conjugated secondary antibodies, and finally to an ECL reagent. Signals were captured on X-ray films and analyzed by densitometry using the ImageJ software. Technical tips used for ensuring intra-gel and inter-gel comparison as well as semi-quantitative analysis of the signals are described in Supplementary Material.

The primary antibodies used were directed against calcineurin (catalytic subunit), calsequestrin type 1 (CSQ1) and type 2 (CSQ2), DIO1, DIO2, DIO3, FoxO1, parvalbumin, PGC-1α, and the sarco-endoplasmic reticulum Ca^2+^-ATPases SERCA1 and SERCA2. Technical details about these antibodies are given in Table 1. Validation of the antibodies used for detecting and quantifying DIO1, DIO2, and DIO3 is shown in Supplementary Material.

**Table 1:** **Primary antibodies used for analysis of protein levels**.

**Target protein**	**Host (clonality^a^)**	**Dilution (competitor^b^)**	**Source**	**Cat. number**
Calcineurin	Rabbit (P)	1:1000 (BSA)	CST^c^	#2614
Calsequestrin 1	Mouse (M)	1:3000 (BSA)	Thermo scientific	MA3-913
Calsequestrin 2	Rabbit (P)	1:2000 (BSA)	Thermo scientific	PA1-913
DIO1	Rabbit (P)	1:1000 (BSA)	Proteintech Europe	11790-1-AP
DIO2	Rabbit (P)	1:200 (milk)	SCB^d^	sc-98716
DIO3	Rabbit (P)	1:1000 (milk)	Novus biologicals	NBP1-05767
FoxO1	Rabbit (M)	1:1000 (BSA)	CST	#2880
Parvalbumin	Mouse (M)	1:2000 (BSA)	Merck-Millipore	MAB1572
PGC-1α	Rabbit (P)	1:1000 (BSA)	Novus biologicals	NBP1-04676
SERCA1	Mouse (M)	1:2000 (BSA)	Thermo scientific	MA3-911
SERCA2	Mouse (M)	1:1000 (BSA)	Abcam	ab3625

a *M, monoclonal; P, polyclonal*.

b *Refers to the blocking reagent used (5%) during the incubation step with the primary antibodies*.

c *Cell Signaling Technology*.

d *Santa Cruz Biotechnology*.

### SERCA activity

The enzymatic activity of the SERCA pumps was measured by an NADH-coupled assay (Warren et al., 1974; Strosova et al., 2011; Viskupicova et al., 2015). Muscle microsomes were prepared from the gastrocnemius muscle of semistarved, refed and controls rats according to Warren et al. (1974), modified by Karlovska et al. (2006). Because of their skeletal muscle origin, microsomes comprised mostly sarcoplasmic reticulum (SR) vesicles, and 70–80% of total protein content was made up of SERCA protein (Lenoir et al., 2002). The sarcoplasmic reticulum-rich vesicles (final concentration 12.5 μg protein/cuvette) were added to the assay mixture (40 mM Hepes pH 7.2, 0.1 M KCl, 5.1 mM MgSO_4_, 2.1 mM ATP, 0.52 mM phosphoenolpyruvate, 1 mM EGTA, 0.15 mM NADH, 7.5 IU of pyruvate kinase, 18 IU of lactate dehydrogenase, pre-incubated at 37°C for 10 min). The reaction was started by addition of CaCl_2_ (final concentration 1 mM). The reaction rate was determined by measuring the decrease in NADH absorbance at 340 nm, at 37°C. Specific SERCA activity (IU/mg; i.e., μmol substrate/min/mg of protein) was calculated using the following equation:

$$\begin{array}{l} \frac{IU}{mg}=\frac{\Delta A_{340nm}\cdot V}{6.22\cdot m}, \end{array}$$

where ΔA_340nm_ represents a change in absorbance at 340 nm per min, V is the volume of the reaction mixture (mL), 6.22.10^3^ L.mol^−1^.cm^−1^ is the absorption coefficient for NADH, and m represents the total amount of protein in the reaction mixture (mg).

### Data analysis and statistics

All data are presented as means ± SEM (*n* = 8–10 rats per group). Direct comparisons between the two experimental groups and their respective control groups, i.e., SS vs. C_SS_ or RF vs. C_RF_, were performed using Mann-Whitney tests, considering that the sample size was too small for assuming Gaussian distribution of the values. The level of statistical significance was set at *P* ≤ 0.05. Statistical analyses were performed and graphs were constructed using GraphPad Prism version 6.01 (GraphPad software Inc., La Jolla, CA, USA).

## Results

### Effects of semistarvation-refeeding on rat body weight and triceps size

In accordance with our earlier observations with this model (Crescenzo et al., 2003; Cettour-Rose et al., 2005; Mainieri et al., 2006), caloric restriction led to growth arrest, whereas isocaloric refeeding led to weight gain at a slightly higher rate than in the control rats. As shown in Table 2, at the end of the 2-week period of caloric restriction, body weights of the semistarved rats and their controls were similar (SS vs. Css: 222 vs. 224 g), and after 1 week of isocaloric refeeding, body weights tended to be higher in the refed than in the control animals (RF vs. CRF: 296 vs. 281; non-significant). The data on the triceps surae muscles are also shown in Table 2. After semistarvation, the triceps were 20.4% bigger than the control ones. As the rats' body weight remained unchanged, this translated into a +19.9% higher triceps specific mass. This increase was due do an enlargement of the muscles, perpendicular to the long axis, as demonstrated by the +21.2% increase in the triceps CSA.

**Table 2:** **Effects of the dietary interventions on body weight and triceps size**.

	**C_SS_**	**SS**	**C_RF_**	**RF**
**RAT**				
Body weight (g)	224±4	222±2^ns^	281±4	296±4^ns^
**TRICEPS**				
Absolute mass (mg)	1467±32	1765±33^***^	1968±36	2217±75^**^
Specific mass (mg/g)	6.65±0.17	7.97±0.11^***^	7.02±0.09	7.50±0.23^ns^
Optimal length (mm)	30.7±0.4	30.5±0.6^ns^	33.8±0.6	36.0±0.7^*^
CSA (mm^2^)	45.2±0.9	54.7±1.3^***^	55.1±1.2	60.5±1.4^*^

* *P ≤ 0.05*;

** *P ≤ 0.01*;

*** *P ≤ 0.001*;

ns *, not significant*,

*comparing SS or RF to their related control. Mann-Whitney test, n = 10*.

The alterations in triceps mass and shape partly persisted after refeeding for 1 week: the absolute mass, and CSA in the refed RF group remained significantly higher than in the C_RF_ control group. Of note, the optimal length required for the triceps to generate the maximum force was increased (+6.7%) after refeeding, underscoring an effect of semistarvation-refeeding on muscle plasticity and visco-elastic properties.

### Effects of semistarvation-refeeding on contractile properties of the triceps surae

**Figure 1:**
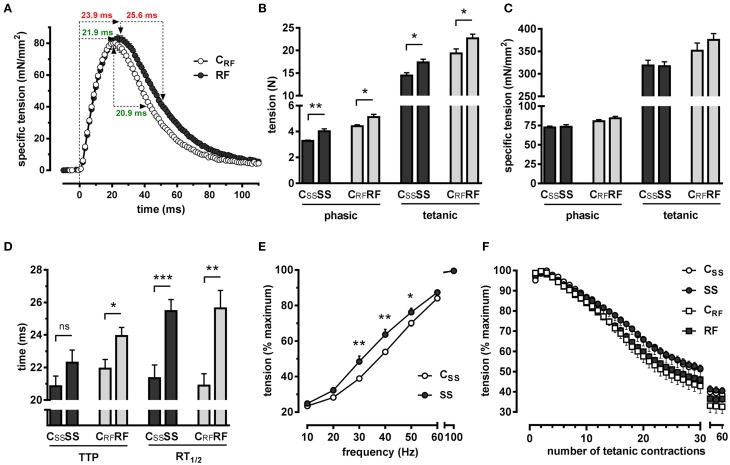
**Electrically-evoked isometric contractions of the triceps surae were recorded ***in situ*** in semistarved and refed rats and in their respective controls**. Force characteristics and kinetics of contraction and relaxation were determined. **(A)** The average phasic traces of refed rats and their controls are depicted, illustrating muscle contraction followed by complete relaxation. The kinetics values (time to peak and time for half-relaxation from the peak) are highlighted in green (control) and in red (refed). **(B)** Absolute phasic and tetanic tensions, **(C)** specific phasic and tetanic tensions, **(D)** kinetics of contraction and relaxation, **(E)** force-frequency relationships, and **(F)** muscle fatigue were determined as described in the text. SS, semistarved rats; C_SS_, control of semistarved rats; RF, refed rats; C_*RF*_, control of refed rats. *n* = 8–10; Mann-Whitney test; ^*^P ≤ 0.05; ^**^P ≤ 0.01; ^***^P ≤ 0.001; ^ns^, not significant, comparing SS or RF to their respective control.

Phasic twitches were recorded after electrical stimulation of the triceps surae from rats after semistarvation for 2 weeks and after refeeding for 1 week. As illustrated in Figure 1A for the C_RF_ and RF groups, the electrical stimulation of the triceps triggered its contraction (rising phase), followed by its complete relaxation (descending phase), allowing to determine phasic tensions (Figures 1B,C), and kinetics of contraction and relaxation (Figure 1D). Then, the stimulation frequency was gradually increased from 10 to 100 Hz and the strongest contraction was used for determining the tetanic tension (Figures 1B,C).

#### Phasic and tetanic tensions

Semistarvation caused the muscle to generate higher phasic (*Po*) and tetanic (*Pt*) absolute tensions (Figure 1B). These effects on force development persisted after refeeding. Importantly, the augmentation of the forces generated by the muscles was not seen any longer after the absolute tensions were transformed into specific tensions (Figure 1C), i.e., after taking into account muscle cross sectional area CSA (Table 2). This revealed that the increase in force was solely due to muscle enlargement and not to a higher performance per surface unit.

#### Kinetics of contraction and relaxation

The dietary interventions caused a marked slowing of the kinetics of contraction and relaxation. This is demonstrated by the increased time required for reaching maximal contraction from baseline (time to peak; TTP) and by the increased time for achieving 50% relaxation from the peak values (time for half-relaxation from the peak; RT_1∕2_) (Figures 1A,D). This slowing, which was evident after semistarvation, became even more pronounced after refeeding. Also, the effects on relaxation were much more pronounced (RT_1∕2_: SS vs. C_SS_: +4.12 ms, *P* ≤ 0.001; RF vs. C_RF_: +4.75 ms, *P* ≤ 0.01) than those on contraction (TTP: SS vs. C_*SS*_: +1.45 ms, *P* = 0.251; RF vs. C_RF_: +2.01 ms, *P* ≤ 0.05).

#### Force-frequency relationship

Force-frequency relationships were determined. The average curves for the C_*SS*_ and SS groups are shown in Figure 1E. The muscle of the SS rats developed a markedly augmented relative force up to 50 Hz when compared to controls, resulting in a clear leftward shift of the force-frequency curve. The control rats required a stimulation frequency of ~38 Hz to develop 50% of the maximum force, whereas the SS rats required only ~30 Hz to reach the same relative force. This is in accordance with the decreased kinetics of contraction and relaxation (Figure 1D). After 1 week of refeeding, the leftward shift of the force-frequency relationship became non-significant (not shown).

#### Fatigability of muscle exposed to repetitive tetanic contractions

We assessed muscle fatigue via a protocol in which muscles are challenged by repetitive tetanizations (Dorchies et al., 2006, 2013; Reutenauer et al., 2008; Reutenauer-Patte et al., 2012). Muscle fatigue was determined at the end of the semistarvation period and after 1 week of refeeding. No difference was found between the groups (Figure 1F; SS vs. C_SS_ or RF vs. C_RF_).

### Effects of semistarvation-refeeding on muscle structure and fiber type composition

No alterations of muscle fiber morphology were found as assessed by hematoxylin-eosin staining of gastrocnemius muscle cross-sections. In particular, no changes in fiber size and in cytochrome C oxidase, lactate dehydrogenase, and succinate dehydrogenase activities were found (data not shown).

**Figure 2:**
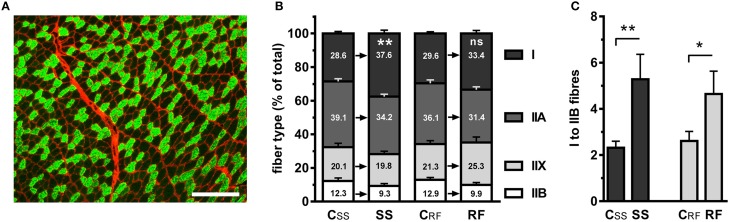
**The distribution of fiber types in gastrocnemius muscles of semistarved and refed rats and their respective controls was determined based on the expression of myosin heavy chain (MyHC) isoforms**. Muscle sections were incubated with monoclonal antibodies to specific MyHC isoforms and fluorescent secondary antibodies. The extracellular matrix was stained with a fluorescent lectin in order to help demarcating the muscle fibers. **(A)** Example of MyHC 1 labeling in the gastrocnemius of a control rat, revealing the slow-twitch, type I fibers (green) and the counterstained extracellular matrix (red). A similar procedure was employed for staining type IIA and type IIB fibers using anti-MyHC 2A and anti-MyHC 2B, respectively. The positive fibers were expressed as the percentage of the total number of fibers. Approximately 1900 fibers were counted on every section. Bar: 100 μm. **(B)** Distribution of fiber types in the experimental groups. The fibers that were not of type I, IIA, or IIB were identified as type IIX. **(C)** Ratio of type I to type IIB fibers. SS, semistarved rats; C_SS_, control of semistarved rats; RF, refed rats; C_RF_, control of refed rats. *n* = 8–10; Mann-Whitney test; ^*^P ≤ 0.05; ^**^P ≤ 0.01; ^ns^, not significant, comparing SS or RF to their respective control.

In order to elucidate the reasons for the slower kinetics of contraction and relaxation, we performed exhaustive fiber typing by labeling the MyHC with isoform-specific antibodies. Figure 2A illustrates the labeling of MyHC type 1. We found that the fibers positive for MyHC 1 were more abundant in the oxidative regions of gastrocnemius muscles after semistarvation, compared to the C_SS_ control rats (+31.5%; *P* ≤ 0.01). MyHC 1 is characteristic for the type I, slow-twitch fibers (Figure 2B). An augmented proportion of type I fibers partly persisted in the gastrocnemius of RF rats (+13.4%), without reaching statistical significance. The accumulation of the type I fibers occurred not only at the expense of the type IIA fibers but also of type IIB fibers and to a lesser extent, of type IIX fibers, demonstrating an overall conversion of fast fibers into slower fibers. As a result, the ratio of type I to IIA fibers was moderately increased (+~51%) but the ratio of type I to IIB fibers was more than doubled (+127%) in the gastrocnemius from SS rats and remained ~78% higher in the RF rats, compared to their respective controls (Figure 2C). The fiber typing data supported our findings on the slower contraction and relaxation kinetics.

### Effects of semistarvation-refeeding on expression levels of slow-twitch muscle markers

The kinetics of contraction and relaxation are partly controlled by the nature and activity of the SERCA pumps. SERCA1 is expressed in fast-twitch fibers (IIB, IIX, IIA), whereas SERCA2 is specific for slow-twitch fibers (type I). SERCA activity was measured in microsomes prepared from the gastrocnemius of the rats. No difference in SERCA activity was found after semistarvation (SS vs. C_SS_) or refeeding (RF vs. C_RF_), although there was a trend toward enhanced activity for both (Table 3).

**Table 3:** **Effects of the dietary interventions on SERCA activity in rat gastrocnemius**.

	**C_SS_**	**SS**	**C_RF_**	**RF**
Activity (IU/mg prot.)	3.39 ± 0.45	3.80 ± 0.34^ns^	3.62 ± 0.34	4.29 ± 0.62^ns^

ns *, not significant*,

*comparing SS or RF to their related control. Mann-Whitney test, n = 10*.

**Figure 3:**
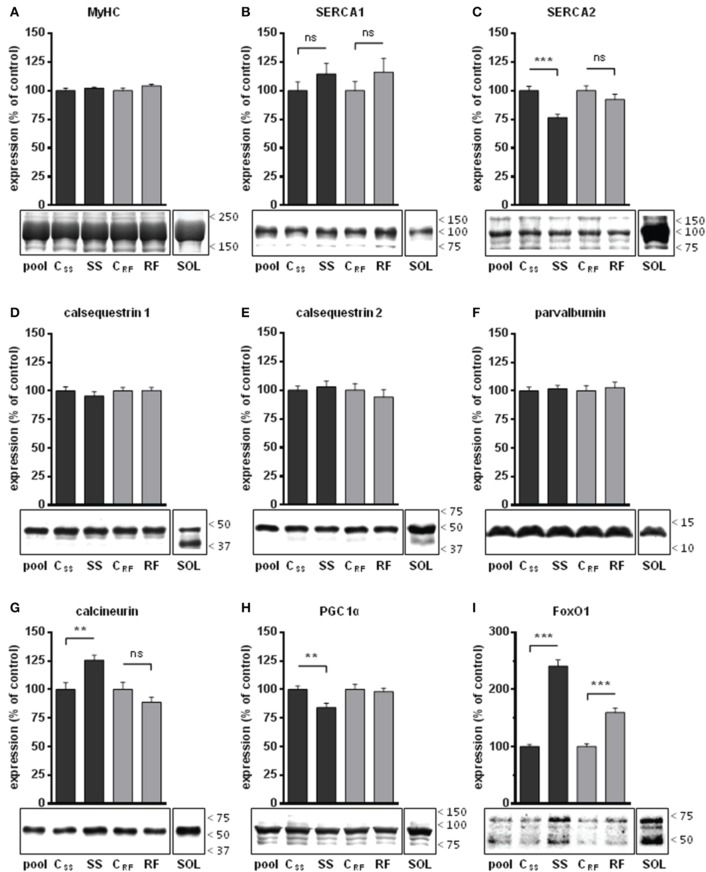
**Expression levels of selected proteins in gastrocnemius extracts from semistarved and refed rats and their respective controls were determined by Western blots**. **(A)** Amount of myosin heavy chains (MyHC) were determined by Coomassie staining in 3 independents experiments. MyHC were similarly abundant in all 4 groups and were thus validated as internal controls for the quantification of the other muscle markers under study. Selected proteins that are well known markers of Ca^2+^ handling in slow-twitch or fast-twitch fibers were analyzed: **(B)** SERCA1, **(C)** SERCA2, **(D)** calsequestrin 1, **(E)** calsequestrin 2, and **(F)** parvalbumin. The abundance of transcription factors that control the development of slow-twitch muscles either positively or negatively were also determined: **(G)** calcineurin, **(H)** PGC1-α (the most intense band at the expected size for the full-length protein was analyzed; the smaller molecular weight bands likely to be degradation products were excluded), and **(I)** FoxO1 (all bands, likely representing native, methylated, and acetylated forms, were quantified). The signals **(B–I)** were corrected for their MyHC content and normalized to the signal of a pool sample (a mixture of aliquots of all extracts) loaded on the gels for the purpose of intra-gel and inter-gel comparison. The signals from an extract of soleus muscle (a slow-twitch muscle), referred to as SOL, are shown for comparison. They were acquired under the same exposure condition as the experimental groups. The position of the molecular weight markers (kDa) is shown on the right side of the blots. SS, semistarved rats; C_SS_, control of semistarved rats; RF, refed rats; C_RF_, control of refed rats. *n* = 10; Mann-Whitney test; ^**^P ≤ 0.01; ^***^P ≤ 0.001; ^ns^, not significant, comparing SS or RF to their respective control.

We investigated whether the slower kinetics of contraction and relaxation were accompanied by alterations in the expression of selected markers of slow *vs*. fast fibers in rat gastrocnemius (Figure 3). The expression levels of SERCA pumps and of the calcium-buffering proteins, CSQ1, CSQ2, and parvalbumin were normalized to that of total MyHC (Figure 3A). The level of SERCA1 (specific for fast muscle fibers) tended to increase after semistarvation and refeeding, although the inter group differences were not significant (Figure 3B). SERCA2, which is specific for type I fibers, found predominantly in slow muscles, was significantly decreased after semistarvation; an effect that was completely reversed by refeeding (Figure 3C). As shown in Figures 3D–F, semistarvation and refeeding had no impact on the abundance of the fast muscle-specific markers CSQ1 and parvalbumin or on the slow muscle-specific marker CSQ2.

The characterization of muscle markers was extended to several transcription factors known to control the acquisition of slow-twitch vs. fast-twitch phenotype, namely calcineurin, PGC1-α, and FoxO1. We found that after semistarvation, the levels of calcineurin were significantly increased (+26%) and those of FoxO1 were more than doubled (+141%), whereas PGC1-α amounts were decreased (−16%) (Figures 3G–I). After refeeding, only the levels of FoxO1 remained elevated (+60%) compared to the control group.

### Effects of semistarvation-refeeding on muscular net T_3_ neogenesis and deiodinase levels

The rate of net T_3_ neogenesis was assessed *ex vivo* in extracts of gastrocnemius muscles from rats after semistarvation and after refeeding. The *de novo* net T_3_ synthesis was found to be significantly lower in muscles of SS rats (−18%), as well as in muscles of RF rats (−14%) relative to their controls (Table 4).

**Table 4:** **Effects of the dietary interventions on net T_3_ neogenesis in rat gastrocnemius muscle**.

	**C_SS_**	**SS**	**C_RF_**	**RF**
T_3_ (pmol/mg prot./min)	0.51 ± 0.02	0.42 ± 0.01^***^	0.43 ± 0.02	0.37 ± 0.01^**^

** *P ≤ 0.01*;

*** *P ≤ 0.001*,

*comparing SS or RF to their related control. Mann-Whitney test, n = 10*.

**Figure 4:**
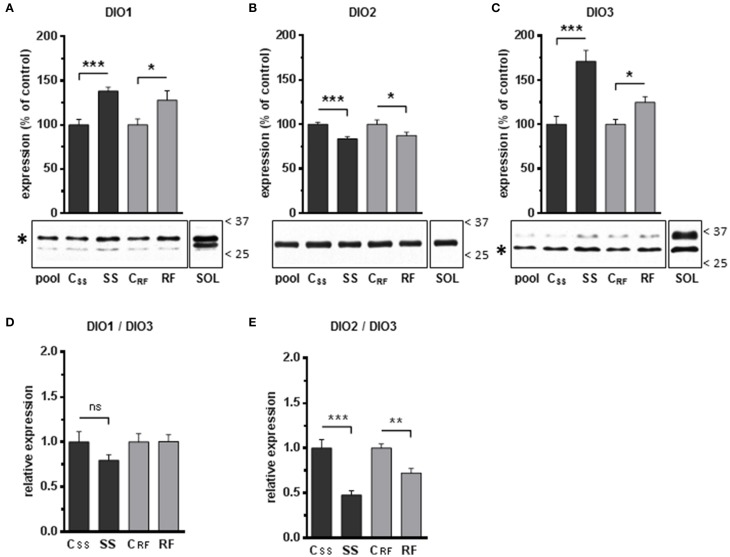
**Expression levels of the iodothyronine deiodinases DIO1, DIO2, and DIO3 in gastrocnemius extracts from semistarved and refed rats and their respective controls were determined by Western blots**. The graphs show the expression levels of **(A)** DIO1, **(B)** DIO2, and **(C)** DIO3. The procedures for the normalization of the signals as well as the definition of the Pool and SOL extracts are described in the Legend to Figure 3. The position of the molecular weight markers (kDa) is shown on the right side of the blots. Asterisks indicate the bands that were identified as DIO1 and DIO3 proteins based on antibody validation experiments (see Supplementary Material for details). The faint extra bands likely represent non-specific labeling of other proteins. The ratios of DIO1-to-DIO3 **(D)**, and DIO2-to-DIO3 **(E)**, were calculated. SS, semistarved rats; C_SS_, control of semistarved rats; RF, refed rats; C_RF_, control of refed rats. *n* = 10; Mann-Whitney test; ^*^P ≤ 0.05; ^**^P ≤ 0.01; ^***^P ≤ 0.001; ^ns^, not significant, comparing SS or RF to their respective control.

Because net T_3_ neogenesis is the result of T_3_ synthesis from T_4_ (through deiodination catalyzed by DIO1 and/or DIO2) and T_3_ degradation by DIO3, we analyzed the expression of the deiodinases DIO1, DIO2 and DIO3 in gastrocnemius muscles (Figure 4). As shown in Supplementary Material, we perform an extensive validation of the antibodies used for Western-blot analysis of DIO expression in rat skeletal muscle. We found that DIO1 and DIO3 were more abundant (+38% and +71%, respectively), whereas DIO2 was less abundant (−16%) in gastrocnemius muscle after semistarvation (Figures 4A–C). These differences were all highly significant and partly persisted after refeeding (+28%, −13%, and +25% for DIO1, DIO2, and DIO3, respectively). As a consequence of these differential changes in DIO levels, the relative DIO1-to-DIO3 ratio was not significantly affected by semistarvation (Figure 4D), whereas the DIO2-to-DIO3 ratio was strongly diminished to ~48% of the control value and was only partly corrected to normal values after refeeding (Figure 4E). These alterations in DIO levels likely accounted for the diminished local net T_3_ neogenesis in gastrocnemius muscles.

## Discussion

It has long been known that patients clinically diagnosed as being malnourished have a reduced rate of skeletal muscle contraction-relaxation (Lopes et al., 1982; Russell et al., 1983b; Chan et al., 1986; Pichard and Jeejeebhoy, 1988; Nishio and Jeejeebhoy, 1991) and that these changes in contractile properties can be induced by caloric restriction or prolonged starvation in humans (Russell et al., 1983a) and rats (Russell et al., 1984a). The focus of these past studies has been in relation to the mechanisms by which malnutrition leads to impaired skeletal muscle mechanical functions and physical disability (Lopes et al., 1982; Pichard and Jeejeebhoy, 1988). However, a lower speed of the contraction–relaxation cycle, by virtue of the associated reductions in ATP turnover (Pichard et al., 1988; Mijan de la Torre et al., 1993), and hence increased muscle mechanical efficiency, can also contribute to the adaptive reduction in thermogenesis that occurs in response to caloric restriction. We report here, that during caloric restriction and refeeding, hindlimb muscles showed delayed contraction-relaxation kinetics, supported by an increased proportion of slow at the expense of fast muscle fibers. From a molecular point of view, we demonstrated that semistarvation-refeeding caused major changes in the muscular expression of transcription factors that control slow *vs*. fast phenotype, and of the deiodinases DIO1, DIO2, and DIO3, which is in agreement with decreased availability of muscular T_3_, the main active thyroid hormone. Collectively, altered thyroid hormone metabolism, fiber type composition and contractile properties constitute mechanisms by which diminished skeletal muscle thermogenesis could contribute to energy conservation during weight loss and weight recovery, and hence contribute to the thrifty metabolism that accelerates fat recovery or catch-up fat during refeeding.

### Consequences of semistarvation-refeeding on muscle contraction, fiber type switch, and calcium handling

#### Slower contraction-relaxation kinetics: Roles of muscle structure and fiber type

The prolonged contraction-relaxation times, assessed as time to peak and time for half-relaxation from the peak in response to electrical stimulation, during caloric restriction and refeeding, were accompanied by changes in muscle fiber type. Specifically, the semistarved and refed groups displayed a slower phenotype than their respective controls. In terms of fuel economy, this would be advantageous as there is evidence that slow-twitch muscles use less ATP per unit of isometric tension than fast-twitch muscles (Wendt and Gibbs, 1973; Crow and Kushmerick, 1982; Henriksson, 1990). Whether this shift from fast-to-slow fiber composition contributes to the prolonged contraction-relaxation times is, however, uncertain. Although, a greater atrophy of fast fibers than slow fibers has often been reported to correlate with delayed contraction-relaxation times in muscle from malnourished rats and humans (Essen et al., 1981; Lopes et al., 1982; Russell et al., 1984a,b; Henriksson, 1990), several studies failed to demonstrate a relationship between the magnitude of changes in relaxation rate and the proportion of fiber types in muscle (Kelsen et al., 1985; Lewis et al., 1986; Sieck et al., 1989), or showed that the alterations identified as the cause of the slowing occurred in both, type I and type II fibers (Nishio and Jeejeebhoy, 1991).

Our data do not fully clarify these controversies. Although we found a clear increase of type I fibers in the red (slow, oxidative) regions of the gastrocnemius after semistarvation, we did not observe a similar fast-to-slow transition in the white (fast, glycolytic) regions of that muscle (not shown). Therefore, it is not clear whether the slower MyHC signature restricted to the slow region is the only cause for the major slowing in contraction and relaxation kinetics observed in response to electrical stimulation.

Skeletal muscle in both semistarved and refed rats showed no differences in the resistance to fatigue compared to their respective controls. This might be explained by the fact that part of the type IIA fibers were converted into type I fibers, both of which being often referred to as fatigue-resistant fibers. The fatigue assay employed is very challenging to muscle fibers. Based on unpublished observations from our laboratory, we believe that the drop in force in this assay is caused by a combination of metabolic exhaustion, secondary to limited nutriment availability and accumulation of inorganic phosphate, as has been described by Allen and colleagues (Allen et al., 2008; Allen and Trajanovska, 2012) and mechanical damage to the fibers. The similar resistance to fatigue of experimental groups further suggests that semistarvation and refeeding did not induce major disturbances in muscle structure.

#### Slower contraction-relaxation kinetics: Roles of calcium handling proteins

The pattern of calcium handling proteins expressed in a given muscle fiber partly controls contraction intensity and shape. Ca^2+^ ions released from the sarcoplasmic reticulum triggers contraction, whereas reuptake of Ca^2+^ into the sarcoplasmic reticulum allows relaxation to take place (Berchtold et al., 2000; Reggiani and te Kronnie, 2006). Altered Ca^2+^ cycling could *a priori* contribute to the increased time to peak observed: excessive Ca^2+^ release from the sarcoplasmic reticulum and inefficient Ca^2+^ reuptake through SERCA pumps would result in prolonged cytosolic calcium transients and increase the period of time until which relaxation could occur. Moreover, a link between thyroid hormone levels and Ca^2+^ handling in skeletal muscle has been described (Everts, 1990; Minatoya et al., 2007). However, our findings do not support impaired intracellular Ca^2+^ cycling. Indeed, when expressed per cross-sectional area, no differences in skeletal muscle force, neither phasic nor tetanic, were observed between the semistarved rats and their controls or between the refed rats and their controls. This suggests that Ca^2+^ cycling was essentially unaffected (Berchtold et al., 2000). Such a contention is consistent with our findings that SERCA activity remained constant after semistarvation and refeeding. It is likely that the decreased SERCA2 levels after semistarvation were compensated by the trend of SERCA1 to increase, resulting in stable SERCA activity overall. The fact that levels of sarcoplasmic reticulum (CSQ1, CSQ2) as well as cytosolic (parvalbumin) Ca^2+^-buffering proteins (Berchtold et al., 2000; Reggiani and te Kronnie, 2006) also remained unchanged, further supports the hypothesis that Ca^2+^ cycling was spared in this model of semistarvation-refeeding.

#### Slower contraction-relaxation kinetics: Roles of myosin heavy and light chains

Muscle relaxation occurs as a consequence of Ca^2+^ removal from the cytosol to concentrations below the myofibrillar activation threshold. In other words, during phasic twitches, Ca^2+^ handling proteins control the time until the onset of relaxation but not the rate at which relaxation occurs (Berchtold et al., 2000; Reggiani and te Kronnie, 2006). The latter is mostly dictated by the nature of the myosins that are expressed in a given muscle. Accordingly, the prolonged time for half-relaxation from the peak could be explained, at least partly, by the increased proportion of type I fibers. Other mechanisms, such as changes in the pattern of myosin light chains and/or their phosphorylation status, may also be involved in the slower relaxation (Edwards et al., 1975; Zhang et al., 2006; Barclay et al., 2007; Westerblad et al., 2010). Indeed, the rate of ATP hydrolysis by the MyHC heads and hence actin-myosin cross-bridge kinetics are mostly controlled by the myosin light chains that are associated to the MyHC heads (Berchtold et al., 2000; Reggiani and te Kronnie, 2006). In support of this, others have shown that the slower relaxation of fatigued mouse fibers was primarily caused by altered actin-myosin cross-bridge properties more than defects in Ca^2+^ handling (Westerblad and Allen, 1993; Westerblad et al., 1997).

#### Slow-contracting muscle but unusual molecular signature: Roles of transcription factors

As evidenced by the analysis of muscle force in sedated rats and by MyHC typing on gastrocnemius sections, semistarvation caused a remarkable slowing of muscle contraction-relaxation kinetics that persisted after refeeding. The *in vivo* functional findings correlated well with the accumulation of type I fibers that are the molecular motors for slow contraction-relaxation cycling. The greater proportion of slow fibers was also in line with the leftward shift of the force-frequency relationship after semistarvation, as low stimulation frequencies were able to recruit larger fractions of the maximum force.

We hypothesized that the slower fiber phenotype would be substantiated by other differences in the expression of slow muscle markers at the expense of those of fast muscle. For instance, we expected a decrease in SERCA1, CSQ1, and parvalbumin, which are typical of fast-twitch muscles and/or an increase in SERCA2 and CSQ2, which are characteristic of slow-twitch muscles (Berchtold et al., 2000; Reggiani and te Kronnie, 2006). However, this was not the case. On the contrary, we found a trend toward an increase of SERCA1 and a significant decrease in SERCA2 after semistarvation, and the levels of the Ca^2+^ buffering proteins CSQ1, CSQ2, and parvalbumin remained constant, regardless of the dietary status. Therefore, although MyHC expression supports the slower muscle behavior observed, the muscles did not exhibit the typical molecular signature that one may expect from slow-contracting muscles.

We propose that this incomplete slow signature resulted from an aberrant expression of several transcription factors that regulate the acquisition and maintenance of slow phenotype in skeletal muscle. For instance, calcineurin and PGC-1α are positive regulators of the slow phenotype and were reported to stimulate fast-to-slow transition (Lin et al., 2002; Vescovo et al., 2005; Mallinson et al., 2009; Jiang et al., 2010; Sakuma and Yamaguchi, 2010). We found that calcineurin was increased after semistarvation, which correlated with the observed slower phenotype. PGC-1α levels, on the contrary, were decreased, which may promote the opposite effects. FoxO1 levels were markedly elevated after semistarvation, which is consistent with previous reports demonstrating FoxO1 accumulation after fasting (Gross et al., 2008). FoxO1 is more abundant in the mixed gastrocnemius muscle than in the slow-twitch soleus muscle (Yuan et al., 2011). By contrast to calcineurin and PGC-1α, FoxO1 acts as a negative regulator of slow muscle fibers (Kamei et al., 2004). In this context, it is worth mentioning that persisting elevated levels of FoxO1 may stimulate the reversion of the slow phenotype by inhibiting calcineurin activity directly (Yuan et al., 2011). Accordingly, the shift of MyHC 1-positive fibers and of SERCA2 levels toward control values during refeeding may result from the persistence of elevated FoxO1 together with normalized amounts of calcineurin and PGC-1α.

We propose that the differential expression of calcineurin, PGC-1α, and FoxO1 affects the expression levels of the proteins coded by their target genes, for example MyHC 1, and that may be the cause for the incomplete slow signature in muscle fibers. Such an unusual molecular pattern may also explain that the even slower contraction-relaxation kinetics after refeeding were associated with a normal force-frequency relationship.

### Muscular levels of deiodinases and kinetics of thyroid hormones during catch-up fat

Whatever the specific intracellular events underlying the slowed contraction-relaxation of skeletal muscle and the associated switch from fast-to-slow fibers during caloric restriction and their persistence during refeeding, it is highly likely that they are modulated by alterations in thyroid hormone availability and actions. On the one hand, the hypothyroid status induced by caloric restriction can be expected to lead to a fast-to-slow fiber transition since hypothyrodism in patients, chemically-induced hypothyroidism in rodents, or deficiency of thyroid hormone receptors in mice, have all been shown to markedly affect muscle fiber proportions in this direction (Ianuzzo et al., 1977; Wiles et al., 1979; Johansson et al., 2003; Simonides and van Hardeveld, 2008). On the other hand, such a status can also lead to higher energetic efficiency, affecting the speed of the contraction–relaxation cycle of skeletal muscle (Wiles et al., 1979; Caiozzo and Haddad, 1996; Everts, 1996). In a previous study (Mainieri et al., 2006) using this rat model of semistarvation-refeeding, we observed that plasma levels of both T_4_ and T_3_ were significantly lower during caloric restriction than in controls, but their restoration kinetics were different. Whereas plasma T_4_ was restored to control levels within 5 days of refeeding, plasma T_3_ remained lower than controls on day 10 of refeeding. The kinetics of T_3_ restoration thus seem to parallel the rate of fat recovery in our rat model. However, thyroid hormone concentrations are known to be affected by local metabolism, such as conversion of T_4_ to the active hormone T_3_ by type 1 and type 2 iodothyronine deiodinases (DIO1 and DIO2, respectively) and inactivation of T_4_ and T_3_ by type 3 iodothyronine deiodinase (DIO3) (Salvatore et al., 2014). In fact, DIO3 decreases T_3_ availability within the skeletal muscle by two ways: it prevents conversion of T_4_ to T_3_ by catalyzing the conversion of T_4_ to reverse T_3_(rT_3_) instead, and it also catalyzes the degradation of T_3_ to 3,3′-T_2_ (Salvatore et al., 2014).

The regulated expression of DIO2 and DIO3 occurs in many tissues, including skeletal muscle, and allows for a tissue-specific modulation of intracellular thyroid hormone activity that is independent on the circulating thyroid hormone levels, thereby increasing the regulatory potential in gene expression (Huang and Bianco, 2008; Salvatore et al., 2014). Thus, in our rat model, the possibility of a lower T_3_ availability in skeletal muscle could be contributed not only from a lower plasma T_3_ levels (Mainieri et al., 2006; Summermatter et al., 2008), but also from altered deiodinase activities. This latter contention would be consistent with our findings here that the kinetics of T_3_ generation in skeletal muscle homogenates incubated with T_4_ were significantly lower after semistarvation and refeeding compared to their respective controls. Additional findings reveal that such reductions in muscle T_3_ availability in our rat model likely result from decreased DIO2, together with increased DIO3, during both, caloric restriction and refeeding.

DIO3 is barely detectable in adult tissues and the cellular compartment in which T_3_ inactivation occurs remains undetermined (Huang and Bianco, 2008). Thus, a role of DIO3 in local tissue modulation of thyroid hormone metabolism in response to weight loss induced by caloric restriction or disease-cachexia has long been unrecognized, despite earlier reports of robust stimulation of DIO3 in various tissues, including skeletal muscle, in hospitalized critically ill patients (Peeters et al., 2003, 2005; Rodriguez-Perez et al., 2008). Furthermore, results from a study in chicken (Van der Geyten et al., 1999) point to the fact that starvation increases liver DIO3 levels by more than 3-fold within 24 h of caloric restriction, and that this increase is associated with a decrease in plasma T_3_ levels. Based on our findings showing marked upregulation of DIO3 expression associated with diminished T_3_ availability in muscle during caloric restriction and weight recovery, diminished intracellular T_3_ availability could be a key factor underlying the shift in fast-to-slow muscle fiber and the slowed kinetics of contraction-relaxation. These changes would contribute to the suppression of thermogenesis, operating as a function of fat store depletion, and hence in the thrifty metabolism that persists during weight recovery to accelerate the restoration of the fat stores.

## Conclusion

The analysis of isometric phasic and tetanic twitches, force-frequency relationship, and fatigue characteristics of leg muscles suggests that the prolonged contraction-relaxation observed during semistarvation persists during the phase of catch-up fat, but is unlikely to be explained by changes in muscle structure or in Ca^2+^ handling. Instead, our data suggest that semistarvation-refeeding causes aberrant expression of transcription factors within skeletal muscle. This stimulates differing signaling pathways, whose integration by the myofibers results in an unusual pattern of expression of slow *vs*. fast muscle proteins. The altered muscle function and, in particular, the slower kinetics of contraction-relaxation, are likely induced by changes in thyroid hormone levels. This is strongly suggested by diminished T_3_ availability within skeletal muscle and supported by a decrease in DIO2 that occurred concomitantly with an increase in DIO3.

## Author contributions

Conceived and designed the experiments: AD, PA, MS, OP, UR, OD, LN. Performed the experiments: PA, OD, MS, OP, DA, LN. Analyzed the data: OD, PA, MS, OP, AD, UR, LN. Contributed reagents/materials/analysis tools: AD, JM, UR, OD, LS. Wrote the paper: AD, OD, PA, LN. Edited the manuscript: UR, JM, MS, DA, OP, LS, LN.

### Conflict of interest statement

The authors declare that the research was conducted in the absence of any commercial or financial relationships that could be construed as a potential conflict of interest.

## Abbreviations

CSA: cross-sectional area
CSQ: calsequestrin
DIO: iodothyronine deiodinase
FoxO1: forkhead box protein O1
MyHC: myosin heavy chain
PGC1-α: peroxisome proliferator-activated receptor gamma coactivator 1-α
RT_1∕2_: time for half-relaxation from the peak
SERCA: sarco-endoplasmic reticulum calcium ATPase
TTP: time to peak.
