# Supplementary material for: Caloric restriction induces energy-sparing alterations in skeletal muscle contraction, fiber composition and local thyroid hormone metabolism that persist during catch-up fat upon refeeding
Source: Front Physiol. 2015 Sep 16;6:254. doi: 10.3389/fphys.2015.00254 (PMC4584973; doi:10.3389/fphys.2015.00254)
Supplement: Supplementary file 1 [file DataSheet1.DOCX]

Supplementary Material

**Caloric restriction induces energy-sparing alterations in skeletal muscle contraction, fiber composition and local thyroid hormone metabolism that persist during catch-up fat upon refeeding**

Paula B.M. de ANDRADE, Laurence A. NEFF, Miriam K. STROSOVA, Denis ARSENIJEVIC, Ophélie PATTHEY-VUADENS, Leonardo SCAPOZZA, Jean-Pierre MONTANI, Urs T. RUEGG, Abdul G. DULLOO^*^, and Olivier M. DORCHIES^*^

***** Corresponding Authors: [abdul.dulloo@unifr.ch](mailto:abdul.dulloo@unifr.ch), [olivier.dorchies@unige.ch](mailto:olivier.dorchies@unige.ch)

**1. Supplementary information about procedures used in this study**

1.1 Supplementary information about *in situ* isometric force recordings in triceps of sedated rats

Isometric force recordings of the triceps surae muscle were performed *in situ* essentially as previously described in mice ([Dorchies et al., 2006](#_ENREF_4);[Reutenauer et al., 2008](#_ENREF_10);[Hibaoui et al., 2011](#_ENREF_6);[Nakae et al., 2012](#_ENREF_7);[Reutenauer-Patte et al., 2012](#_ENREF_9);[Dorchies et al., 2013](#_ENREF_3)). Rats were anesthetized by injections of a mixture of urethane (1.5 g/kg, i.p.) and diazepam (5 mg/kg, i.p.). The Achilles tendon of the right hindlimb was exposed and linked to a force transducer. The knee joint was firmly immobilized. Two fine steel electrodes were inserted into the triceps surae muscle. The rats were maintained under a heating lamp throughout the experiment in order to keep body temperature around 37°C. Square wave pulses were delivered via a stimulus controller. Stimulation voltage and muscle length were adjusted to obtain maximum isometric twitch force. The optimal muscle length was determined. A series of 5 phasic twitches was then recorded and the absolute peak twitch tension (Pt), the time to peak (TTP), and the time for half-relaxation from peak (RT1/2) were determined from the average traces. After a 3-min pause, muscles were subjected to a tetanization assay using 200-ms bursts of increasing frequency (from 20 to 100 Hz in increments of 10 Hz) with one burst every 30 s. The successive tensions were used to construct force-frequency curves. The strongest response (usually obtained at 90 or 100 Hz) was taken as the absolute optimal tetanic tension (Po). Finally, after another 3-min pause, muscles were submitted to a fatigue assay for 5 min: frequency was set to 60 Hz, and 60 stimuli were delivered, each consisting of a 2-s train of tetanic stimulation and a 3-s rest. The maximal tension was usually obtained during the first five stimulations. The amplitude of the response then decreased as the stimuli were repeated. The residual tetanic tension was expressed as percentage of the maximum response. Absolute phasic and tetanic tensions (in mN) were converted into specific tensions (in mN per mm^2^) after normalization for the muscle cross-sectional area (CSA). The CSA (in mm^2^) was determined by dividing the triceps surae muscle mass (in mg) by the product of optimal muscle length (in mm) and d, the density of mammalian skeletal muscle (d = 1.06 mg.mm^3^). Control rats were always measured on a same day as their semistarved and refed counterparts.

1.2 Supplementary information about immuno-labeling of myosin heavy chains

Muscle sections were prepared on an HM60 cryostat (Microm) and stored at -80°C until processed as described previously ([Dorchies et al., 2013](#_ENREF_3)). Sections were rehydrated for 5 min in phosphate buffered saline (PBS) and blocked with 3% BSA in PBS for 1 hour. They were then incubated for 1.5 hours with primary antibodies against specific MyHC diluted in washing buffer (0.5% BSA and 0.05% Tween 20). The following monoclonal primary antibodies (purified from hybridomas obtained from the Developmental Study Hybridoma Bank) were used: BA‑D5 (anti-MyHC 1, IgG; 1:2000), SC-71 (anti-MyHC 2A, IgG; 1:1000), BF-F3 (anti-MyHC 2B, IgM; 1:100). Then, the slides were washed 3 times in washing buffer and incubated for 1 hour with fluorescent secondary antibodies diluted in washing buffer. MyHC 1 and 2A were detected with a goat anti-mouse IgG antibody conjugated to Alexa Fluor® 488 (AF_488_) diluted 1:2000 and 1:1000, respectively, in combination with AF_594_-conjugated wheat germ agglutinin (1:200) to stain the extracellular matrix. MyHC 2B was detected with a goat anti-mouse IgM antibody conjugated to AF_594_ (1:100), together with staining of the extracellular matrix with AF_488_-conjugated wheat germ agglutinin (1:1000). Finally, after extensive washing, the nuclei were stained for 5 min in Hoechst dye #33258 (bis-benzimide, 1 µg/mL) in PBS and mounted in Mowiol® 4‑88 (Calbiochem).

1.3 Supplementary information about semi-quantitative determination of proteins by Western blot

In order to allow intra-gel and inter-gel comparison and semi-quantitative analysis of the signals, the following procedure was applied, essentially as described previously ([Reutenauer-Patte et al., 2012](#_ENREF_9);[Dorchies et al., 2013](#_ENREF_3): (i) for each muscle protein to be quantified, 4 gels were run simultaneously and processed in parallel, (ii) the 40 samples were loaded as 10 quadruplets, each consisting of extracts from a C_SS_, a SS, a C_RF_, and a RF rat (iii) a reference extract (Pool), consisting of a mixture of all 40 gastrocnemius extracts, was loaded at both sides of each of the 10 quadruplets, (iv) the portions of the gels that contained the protein of interest were transferred onto a single nitrocellulose membrane, ensuring that all 40 samples and flanking Pool extracts were simultaneously exposed to the blocking solution, primary antibodies, secondary HRP-conjugated secondary antibody, ECL reagent, and X-ray film. An extract of soleus muscle was loaded next to 2 of the sample quadruplets to serve as a positive control for proteins specific of slow-twitch muscle. Signals were first corrected for their total MyHC content calculated from 3 independent determinations on Coomassie-stained gels. The resulting values were normalized to that of the flanking Pool samples. The Pool sample being a mixture of all samples to be compared, this ensured that its signal had an average, non-saturating, intensity. Finally, the values were expressed as the percentage of the control group.

**2. Supplementary Data: validation of the antibodies used for immunoblotting of deiodinases**

Our study demonstrated that semistarvation-refeeding alters the expression levels of the deiodinases DIO1, DIO2, and DIO3 at the protein level in skeletal muscles of rats. In order to confirm these data, we performed additional experiments aiming at validating the antibodies that we used for Western blot detection of the DIO proteins. In brief, we cloned the cDNAs encoding *DIO1*, *DIO2*, and *DIO3* of rat origin in expression vectors, in the presence or absence of a SECIS (selenocysteine insertion sequence) element. The vectors were used for transfecting HEK cells and the extracts were analyzed by Western blot. We were able to confirm over-expression of the DIOs and to demonstrate that the over-expressed signals correlated with the endogenous signals obtained with skeletal muscle extracts. We concluded that the data obtained with the antibodies selected for DIO detection truly represent DIO levels in rat skeletal muscles.

2.1 Sequence analyses for identifying structural elements of rat deiodinases

The sequences used for this work had the following NCBI accession numbers (all from Rattus norvegicus): NM_021653.3 (rat *DIO1* mRNA), NM_031720.3 (rat *DIO2* mRNA), and NM_017210.3 (rat *DIO3* mRNA). The sequence gi|666180875:15680418-15706018, containing the entirety of rat *DIO2* gene sequence, including the long 3’-UTR sequence was retrieved from the NCBI Reference sequence NW_007905843.1.

DIOs are selenoproteins, i.e. proteins that contain the rare amino acid selenocysteine. Their coding sequences (cds) exhibit internal UGA codons, which serve for incorporating selenocysteine residues instead of signalling termination of translation. This atypical use of UGA codons is dependent on the presence of a SECIS element located in the 3’-UTR region of the *DIO* RNAs.

The SECIS of rat *DIO1* and rat *DIO2* were identified using the SelenoDB website (<http://www.selenodb.org/>; accession numbers SPS00001092_2.0 and SPS00001093_2.0). The SECIS sequence of rat *DIO1* and *DIO2* are located ~0.8 kbp and ~4.6 kbp downstream of the cds, respectively. The SECIS of rat *DIO3* was not available in the SelenoDB website and we could not find its sequence on Web ressources. We used the *DIO3* SECIS sequence from Mus musculus (<http://www.selenodb.org/>; accession number SPS00000720_2.0) to probe the sequence NM_017210.3, which contains the full sequence of rat *DIO3* mRNA. We identified the putative rat *DIO3* SECIS sequence based on homology with the mouse homolog (Supplementary Figure 1). The putative SECIS sequence of rat *DIO3* is 72 bp long and is 97% identical (70/72 bp) to the murine homolog. It spans nucleotides 1445-1516 (numbering from the ATG initiation codon) and is located ~0.6 kbp downstream of the cds.

2.2 Cloning of rat deiodinases

Two male Wistar rats (12 weeks of age) were euthanized under deep anaesthesia as described in the main text. Thyroid, interscapular brown adipose tissue, cerebral cortex, liver and gastrocnemius were dissected and snap frozen at -80°C. The tissues were grounded to a fine powder in a mortar cooled in liquid nitrogen. Total RNA were extracted from around 10 mg of each tissue and 1 μg of RNA was reversed transcribed to cDNA as described previously ([Dorchies et al., 2013](#_ENREF_3)).

Primers were designed for PCR-amplification of: (i) the wild type (wt) cDNAs containing the coding sequences (cds) and the entire 3’‑UTR, (ii) the coding sequences (cds) only, (iii) the SECIS sequences of rat *DIO1*, *DIO2*, and *DIO3* (Supplementary Figure 2), using the Q5 high-fidelity DNA polymerase Master mix (New England Biolabs, Bioconcept, Allschwil, Switzerland). The primer sequences are shown in Supplementary Table 1.

A PacI site was included in primers P1, overlapping the ATG initiation codon, and a NheI site was included in primers P2, located just downstream of the SECIS. *DIO1wt* (1650 bp) and *DIO3wt* (1580 bp) were readily amplified by PCR using primers P1-P2 from thyroid and cerebral cortex, respectively. However, we failed to amplify *DIO2wt* (5562 bp) as a single amplicon or by merging 2 shorter PCR products using internal primers, using any of the 5 selected tissues. The program RNAfold (<http://rna.tbi.univie.ac.at/cgi-bin/RNAfold.cgi>) revealed the presence of numerous secondary structures in the denatured ssDNA, which likely hampered DNA polymerase progression.

As an alternative approach, we chose to construct chimeric *DIO* cDNAs by coupling any of the 3 SECIS elements downstream of any of the 3 cds. To this aim, we used primers P3, overlapping the terminal stop codon and containing a NheI site, together with primers P1 for generating rat *DIO1cds*, *DIO2cds*, and *DIO3cds* from thyroid, brown adipose tissue, and cerebral cortex, respectively. Primers P4, containing a NheI site, were designed to anneal ~150 bp upstream of the SECIS elements and were used in combination with primers P2 for amplifying the SECIS elements from the same tissues.

The recipient plasmid *ln074* (unpublished data) was based on pSMD2sc-U7DTex23, a self-complementary adeno-associated virus (scAAV) expression vector kindly donated by Dr. Aurélie Goyenvalle (University of Versailles-Saint-Quentin, France), and similar to pAAV-U7-SD23/BP22 published earlier ([Goyenvalle et al., 2004](#_ENREF_5)). For constructing the plasmid *ln074*, the XbaI-NcoI insert of pSMD2sc-U7DTex23 was excised and replaced by a multiple cloning site containing restriction sites for XbaI, AscI, AsiSI, FseI, PacI, NheI, and Bsu36I, a synthetic polyadenylation signal and finally restriction sites for BamHI and NcoI. A CMV promoter was inserted into AsiSI and FseI sites, and an EGFP reporter gene was inserted into PacI and NheI sites.

PCR fragments *DIO1wt*, *DIO3wt*, *DIO1cds*, *DIO2cds*, and *DIO3cds* were digested by PacI and NheI and ligated into PacI-NheI digested *ln074*. Then, the plasmids bearing *DIO1cds*, *DIO2cds*, and *DIO3cds* were linearized with NheI and ligated with NheI-digested P4-P2 PCR fragments containing any of the SECIS elements. Proper orientation of the SECIS elements was verified by PCR and sequencing. As illustrated in Supplementary Figure 2, 9 chimeric *DIOs* were generated, each containing one of the 3 SECIS located around 250 bp downstream of any of the 3 *DIO* cds. They were named by juxtaposing the cds and SECIS they derived from in the form “cds.SECIS” as described in Supplementary Figure 2. The 14 *DIO* constructs were validated by sequencing.

Plasmids were prepared using the NucleoBond PC 100 midiprep kit (Macherey-Nagel, Oensingen, Switzerland) according to the manufacturer’s instructions and stored at +4°C until used.

2.3 Transfection of HEK cells with expression vectors encoding rat deiodinases

293T HEK cells were cultured in DMEM supplemented with 10% fetal bovine serum and 10 μg/mL ciprofloxacin in 100‑mm diameter Petri dishes. When the cultures were 40-50% confluent, the cells were transfected with any of the *DIO* plasmids or the *ln074* plasmid expressing EGFP (1 μg/cm^2^) by calcium phosphate precipitation according to standard procedures. Sodium selenite (NaSe, 100 nM) was added to the transfected cultures. Controls included non-transfected cells in which NaSe was omitted and non-transfected cells treated with NaSe. As judged by EGFP fluorescence 24h later, transfection efficacy was around 50% in 2 independent experiments. Variable cell toxicity was observed in cultures transfected with the SECIS-containing constructs (expressing wt versions of *DIO1* and *DIO3* as well as chimeric *DIOs*) whereas the cultures transfected with the *DIO* cds only showed no or limited toxicity similar to the EGFP control. This suggested that the presence of a SECIS element resulted in the expression of functional DIOs that impacted cell phenotype and survival.

2.4 Western-blot analysis of over-expressing extracts

Cell extracts were prepared 36h post-transfection. In brief, cells were washed 2 times with phosphate buffered saline (PBS) at room temperature for preventing cell detachment. Then cells were scrapped on ice in chilled RIPA buffer (PBS containing 1% NP-40 substitute, 0.5% sodium deoxycholate, 0.1% SDS, and 0.2% protease inhibitor cocktail (Sigma Aldrich, Buchs, Switzerland)). After gentle shaking on a rotator at 4°C for 20 minutes, the homogenates were sonicated (10 sec at 4°C) and centrifuged (10000x *g* for 10 min at 4°C). The supernatants were collected and protein content was assayed (BC assay, Thermo Scientific, Reinach, Switzerland). All extracts were adjusted at 2.0 μg/mL in Laemmli buffer containing 100 mM DTT.

We verified by Western-blotting that the constructs led to proper over-expression of the DIOs. In order to visualize a wide range of molecular weights, the extracts (30 μg each) were separated by SDS-PAGE on home-made gradient gels containing 15% acrylamide and 10% glycerol at the bottom and 8% acrylamide at the top. The electrophoresis and blotting procedures were otherwise similar to those described in the main text. Four gels were prepared. One gel was stained with Coomassie Brilliant Blue in order to verify overall sample quality and equal loading. The other gels were blotted onto nitrocellulose membranes, probed with any of the rabbit polyclonal antibodies for DIO1, DIO2, and DIO3 (see main text for references and working dilutions), and signals revealed by chemiluminescence. The gels used for detecting over-expressed DIO1 and DIO3 were stripped and re-probed with mouse monoclonal antibodies (1:5000 in Tris buffered saline-Tween 20 (TBS‑T) containing 5% BSA) against GFP (clone B‑2, sc-9996, Santa Cruz Biotechnology), and α-tubulin (clone DM1A, T9026, Sigma-Aldrich), respectively, followed by an HRP-conjugated goat anti-mouse antibody (1:10000 in TBS‑T containing 5% milk) (BioRad Laboratories, Cressier, Switzerland).

As illustrated in Supplementary Figure 3, Coomassie staining showed that all extracts had similar quality. An extra band was barely observed at around 27 kDa in cells transfected with plasmid *ln074*, which corresponded to over-expressed EGFP. Over-expressed EGFP was readily detected by the anti-GFP antibody. Transfection with plasmids coding for wt, cds, or chimeric versions of rat *DIO1*, *DIO2*, and *DIO3* did not result in visible extra bands in Coomassie stained gel, suggesting moderate over-expression of the DIOs.

After transfection with the plasmids coding for every single *DIO*, being the wt version when available or the chimeric *DIOs*, Western-blot analysis revealed bands at the expected size for the full length proteins (~29 kDa for DIO1 and DIO2, ~34 kDa for DIO3; shown by asterisks in Supplementary Figure 3). The DIO1 antibody detected shorter proteins of apparent molecular weight ~18-20 kDa. Because this band was absent from the DIOcds extract, we concluded that it did not correspond to a truncated form that would not have incorporated selenocysteine, but to a degradation product instead. Similarly, the antibody against DIO3 detected a degradation product of ~22 kDa in extracts over-expressing DIO3.

The antibody against DIO2 showed faint non-specific labelling of proteins of ~50 kDa, which might result from cross-reaction with immunoglobulin heavy chains from the FBS. The antibody against DIO3 labelled a protein of ~70 kDa, which might be albumin from FBS. Toxicity of the over-expressed DIOs caused the detachment of a significant fraction of the cells, which may have resulted in differential washout of FBS during brief washes with PBS.

No signal was obtained from non-transfected cells, suggesting that the endogenous levels of DIOs in 293T HEK cells was very low or that the antibodies did not efficaciously bind human DIOs.

From these data, we concluded that the constructs caused efficient over-expression of the full length DIOs in the presence of sodium selenite and that the antibodies were able to detect over-expressed DIOs in a specific manner. In addition, our experiments revealed that the SECIS elements are exchangeable: the SECIS from any DIO can drive the incorporation of selenocysteine in the cds of another DIO in an efficient manner.

Transfection with the plasmids coding for the cds domains only might have led to over-expression of shorter DIOs, whose translation would be interrupted at the first of the internal UGA codons. The DIO1 polyclonal serum was raised against an immunogenic region that spans over the internal UGA codon. Therefore, depending on the actual epitope, antibodies in the polyclonal mixture may or may not detect the N-terminal truncated form. In our case, only the full length protein was detected, so we conclude that the strongest antibodies are mapping to the region downstream of the selenocystein, or that the truncated proteins, if expressed, are unstable. Theoretically, the DIO2 polyclonal serum, which was raised against a large N-terminal portion of the protein upstream of the first internal UGA codon, might detect both truncated and full length protein. However, no signal was obtained in cells transfected with *DIO2cds*. We conclude that the absence of a SECIS element led to either low DIO2 expression and/or to the expression of an unstable N-terminal protein fragment. The DIO3 antibody was raised against the C-terminal part of the protein, downstream of the selenocysteine insertion site. Therefore DIO3 antibody can detect full-length DIO3 only.

2.5 Comparison of over-expressing extracts with skeletal muscle extracts

Signals obtained with extracts from HEK cells transfected with plasmids bearing *DIO1wt*, *DIO2.2*, and *DIO3wt* were compared to those from skeletal muscle extracts. Serial dilutions of over-expressing extracts were loaded side by side to a representative rat gastrocnemius extract (“Pool” extract, see main text for details) and a soleus muscle extract, used as a positive control for most proteins that we analysed in this study (see Figures 3 and 4 in the manuscript and Paragraph 1.3 of Supplementary material above).

Samples (10 μg of non-transfected cells, 1.5 to 0.15 μg of over-expressing extracts; 30 μg of skeletal muscle extracts) were loaded on a 12% polyacrylamide gel, resolved by SDS-PAGE, and transferred onto nitrocellulose as above. Western-blotting revealed that the signals of over-expressed DIO1, DIO2, and DIO3 had sizes similar to those of the endogenous signals observed in gastrocnemius and soleus muscle extracts, strongly suggesting identity of the proteins in both skeletal muscles and over-expressing cells.

As low as 0.5-0.15 μg of over-expressing extracts resulted in similar signals as 30 μg of muscle extracts. Compared to the experiment shown in Supplementary Figure 3, extra non-specific faint bands were seen at ~37 kDa in some samples with both DIO1 and DIO2 antibodies. This may be due to the longer exposure time required because of the much lower load of over-expressing extracts.

The blots used for detecting DIO1 and DIO3 were stripped and re-probed with antibodies to pan sarcomeric α‑actinin (mouse monoclonal EA-53, A7732, Sigma-Aldrich) used at 1:2000 in TBS‑T containing 5% BSA, and α‑tubulin used as above, respectively. As expected, α‑tubulin was abundant in HEK cell extracts and present in trace amounts in muscle extracts. Conversely, α‑actinin, a skeletal muscle marker, was present in muscle extracts and not in HEK cells.

2.6 Conclusions: expression of DIOs in properly extracted rat skeletal muscle

We concluded from the experiments described above that the signals obtained with the selected antibodies truly represent DIO1, DIO2, and DIO3, respectively. In fact, many studies have previously reported that DIOs are expressed in skeletal muscle, based on RNA or protein expression, or on their enzymatic activities ([Salvatore et al., 1996](#_ENREF_11);[Peeters et al., 2003](#_ENREF_8);[Salvatore et al., 2014](#_ENREF_12)). DIO2, in particular is known to be expressed in skeletal muscle and heart ([Salvatore et al., 1996](#_ENREF_11)). Some commercial sources of antibodies, such as Santa Cruz Biotechnology, even recommend using skeletal muscle as a positive control for DIO2.

Failure to detect a skeletal muscle protein by Western blotting may not truly reflect the absence of that protein from the tissue, but absence of the protein from the extract instead. In fact, skeletal muscle is a very tough and highly organized tissue that is notoriously difficult to homogenize and extract proteins from. Proteins can be readily extracted from most tissues with normo-osmotic buffers with or without low amounts of mild detergents. By contrast, preparing representative protein extracts from skeletal muscle and heart requires specific procedures.

In fact, around half of total muscle proteins are made of sarcomeric proteins such as actin, myosins, and many other associated proteins. These proteins are tightly bound to each other through multiple electrostatic interactions. Homogenisation buffers that have insufficient ionic strength lead to limited solubilisation of such muscle proteins. Moreover, extracted sarcomeric proteins tend to self-assemble into insoluble myofibrillar structures when ionic strength drops (e.g. upon dilution of the sample with low-salt buffer) or at low temperature. Actually, this property has been used for decades for preparing crude myofibrillar material from skeletal muscle (see for instance ([Agbulut et al., 1996](#_ENREF_1);[Agbulut et al., 2003](#_ENREF_2)). As a result, loss of sarcomeric proteins is accompanied by loss of many partners that are linked to them, either directly or indirectly. It is not clear whether DIOs, which are membrane-spanning enzymes, are somehow linked to sarcomeric structures in skeletal muscles, but this cannot be ruled out.

In our study, as in previous studies from our group that required enzymatic assays or Western-blot analysis of skeletal muscle and heart ([Reutenauer-Patte et al., 2012](#_ENREF_9);[Dorchies et al., 2013](#_ENREF_3)) (and manuscripts in preparation in our group by Dorchies et al., Ismail et al., and Neff et al.), the muscle samples were ground in liquid nitrogen to optimize access of the structures to the buffer. Pulverized muscle tissues were extracted in Guba-Straub buffer. This buffer has a very high ionic strength (>400 mM) and contains sodium pyrophosphate, both features ensuring depolymerisation of the myofibrils. Complete solubilisation of muscle components was enhanced by the presence of 0.1% 2‑mercaptoethanol, exposure to ultrasounds (sonication) for 2 times 10 s, followed by addition of 1% Triton X‑100.

In spite of these precautions, subsequent high speed centrifugation usually results in 3 phases: a small whitish pellet presumably made of insoluble cross-linked collagen with large tissue debris above it, a clear top phase containing soluble proteins, and a milky, turbid, intermediate phase that contains partly reassembled myofibrillar components and associated proteins. At this stage, it is important that both the top and intermediate layers are collected and thoroughly mixed, leaving only the insoluble pellet.

The myofibrillar nature of the muscle extracts may also result in protein loss even after dilution in reducing Laemmli buffer and boiling. In fact, during electrophoresis, myosins and maybe also other proteins tend to interact with each other, resulting in poor penetration into the gel in some settings. In order to decrease interactions between sarcomeric proteins during electrophoresis, 2‑mercaptoethanol (10 mM) was added to the cathode chamber.

We believe that these technical tips may explain discrepancies between our findings and previous work by others, who may have failed to detect DIO proteins in skeletal muscle tissues.

# 3. Supplementary Figures and Tables

## 3.1 Supplementary Figures

**Supplementary Figure 1**

**Sequence of putative SECIS element of rat *DIO3***

**Legend to Supplementary Figure 1**

Unexpectedly, the sequence of rat DIO3 SECIS element could not be found on any Web ressource. Aligning the sequence NM_017210.3, which contains the full sequence of rat *DIO3* mRNA, with the sequence of mouse *DIO3* SECIS element (<http://www.selenodb.org/>; accession number SPS00000720_2.0), allowed identification of the putative rat *DIO3* SECIS sequence. The latter is 72 bp long, shares 97% identity (70/72 bp) with the murine homolog, and is located ~0.6 kbp downstream of the coding sequence (nucleotides 1445 to 1516 from the ATG initiation codon).

**Supplementary Figure 2**

**Schematic representation of the rat DIO constructs**

**Legend to Supplementary Figure 2**

**(A)** Schematic representation of the mRNAs of rat DIO1, DIO2, and DIO3, showing the coding sequence (cds) and the 3’‑UTR containing the SECIS element. The location of the primers (P1, P2, P3 and P4) modified to bear restriction sites for PacI and NheI and used for PCR amplification of the wild type DIOs, the cds, or the SECIS elements is illustrated next to DIO1wt. The ~5.5 kbp DIO2wt could not be amplified by PCR, likely because of high content of secondary structures in the mRNA. This prompted the construction of chimeric mini-DIO cDNAs **(B)**, in which the SECIS element of any DIO was cloned downstream of each DIO cds. Values above the cDNAs indicate nucleotides numbered from the ATG initiation codon. The location of the TGA codons used for selenocysteine incorporation are shown by vertical bars across the cDNAs and their position are indicated by values below the cDNAs.

**Supplementary Figure 3**

**Western blot analysis of over-expressed rat deiodinases**

**Legend to Supplementary Figure 3**

293T HEK cells were transfected with expression plasmids bearing wild type versions of *DIO1* or *DIO3*, coding regions (cds) of *DIO1*, *DIO2*, or *DIO3*, any of 9 chimeric *DIOs* containing a cds fused to a SECIS element, or EGFP as a reporter gene. Transfected cells and a non-transfected control were treated with 100 nM sodium selenite (NaSe) to enhance selenocysteine synthesis and incorporation into DIOs. A non-transfected non-treated control was also included. Cell extracts (30 μg) were resolved by SDS-PAGE (8-15% acrylamide gradient gels) and analyzed by Western-blot. **(A)** Coomassie-stained gel showing overall extract quality and equal protein loading. **(B‑D)** Detection of over-expressed DIO1 **(B)**, DIO2 **(C)**, and DIO3 **(D)**. The full-length proteins are indicated by an asterisk. The position of the molecular weight markers (kDa) is shown on the right side of the blots. Extra bands of lower molecular weight in DIO1 and DIO3 over-expressing cells were putatively identified as degradation products. Faint signals at around 50 kDa and 70 kDa in DIO2 and DIO3 over-expressing cells may represent non-specific cross-reaction with other proteins. **(E)** Detection of over-expressed EGFP after stripping of the DIO1 blot. (F) Detection of α‑tubulin, used as a control for equal protein loading and transfer, after stripping of the DIO3 blot.

**Supplementary Figure 4**

**Western blot comparison of rat deiodinases in over-expressing cells and muscle extracts**

**Legend to Supplementary Figure 4**

293T HEK cells were transfected with expression plasmids bearing wild type versions of *DIO1* or *DIO3* or the chimeric *DIO2.2* cDNA. Signals obtained from serial dilutions of DIO over-expressing HEK cell extracts were compared to those from skeletal muscle extracts: a representative rat gastrocnemius extract and a soleus muscle extract, used as a positive control (see Figures 3 and 4 in the manuscript for details). Samples (10 μg of non-transfected cell extract, 1.5, 0.5, and 0.15 μg of over-expressing extracts, 30 μg of skeletal muscle extracts) were resolved by SDS-PAGE on a 12% polyacrylamide gel. **(A)** Ponceau Red staining of a membrane after blotting showing decreasing load of HEK over-expressing extracts in parallel to high load of muscle extracts. **(B-D)**The signals of over-expressed DIO1 **(B)**, DIO2 **(C)**, and DIO3 **(D)**, (shown by asterisks) had the same size as bands in the muscle extracts, which were quantified in our study. Extra non-specific faint bands at ~37 kDa in some samples with both DIO1 and DIO2 antibodies may be due to the long exposure time required because of the low load of over-expressing extracts. **(E)** α‑tubulin in control cells and in HEK cells that over-express DIOs. **(F)** α‑actinin expression in skeletal muscle extracts. The position of the molecular weight markers (kDa) is shown on the right side of the blots.Note that compared to the experiment shown in Supplementary Figure 3 bands may seem fuzzier because a regular 12% acrylamide gel was used instead of a gradient gel, which allows better focusing of the proteins.

## 3.2 Supplementary Tables

**Supplementary Table 1**

*DIO1* P1 5’-ctgagttaattaagccaccATGgggctgtcccagctatg-3’

P2 5’-cgacgtcgctagcctgatggattttaatcgttagc-3’

P3 5’-tgcatgctagcCTAgaactgaggcatgtgtccagg-3’

P4 5’-gacttgctagcccaaatcacaggcgccag-3’

*DIO2* P1 5’-ctgagttaattaagccaccATGggactcctcagcgtagacttg-3’

P2 5’-acattcgctagccctccttgctaaggagacagagatg-3’

P3 5’-catgcatgctagcccagCTAatctagaattcatctcttgctg-3’

P4 5’-catgcgctagcactttataaccaaagcaatcaaaaggtc-3’

*DIO3* P1 5’-gagttaattaagccaccATGcctcgccaggccg-3’

P2 5’-cgatcgatcgctagcactttccccctcttactttagg-3’

P3 5’-gcatgcatgctagcttTTAgagtcgacgtggcctagtacc-3’

P4 5’-gatcgatcgctagctagttgcctggcaccc-3’

**Primers used for PCR amplification of rat deiodinase coding sequences and SECIS elements.**

The PacI restriction site in primers P1 is boxed. The NheI restriction site in primers P2, P3, and P4 is underlined. The ATG initiation codon in primers P1 (forward) and the stop codon in primers P3 (reverse orientation) are shown in uppercase letters.

# 4. Supplementary References

Agbulut, O., Li, Z., Mouly, V., and Butler-Browne, G.S. (1996). Analysis of skeletal and cardiac muscle from desmin knock-out and normal mice by high resolution separation of myosin heavy-chain isoforms. *Biol. Cell* 88**,** 131-135.

Agbulut, O., Noirez, P., Beaumont, F., and Butler-Browne, G. (2003). Myosin heavy chain isoforms in postnatal muscle development of mice. *Biol. Cell* 95**,** 399-406.

Dorchies, O.M., Reutenauer-Patte, J., Dahmane, E., Ismail, H.M., Petermann, O., Patthey- Vuadens, O., Comyn, S.A., Gayi, E., Piacenza, T., Handa, R.J., Decosterd, L.A., and Ruegg, U.T. (2013). The anticancer drug tamoxifen counteracts the pathology in a mouse model of duchenne muscular dystrophy. *Am. J. Pathol.* 182**,** 485-504.

Dorchies, O.M., Wagner, S., Vuadens, O., Waldhauser, K., Buetler, T.M., Kucera, P., and Ruegg, U.T. (2006). Green tea extract and its major polyphenol (-)-epigallocatechin gallate improve muscle function in a mouse model for Duchenne muscular dystrophy. *Am. J. Physiol. Cell Physiol.* 290**,** C616-625.

Goyenvalle, A., Vulin, A., Fougerousse, F., Leturcq, F., Kaplan, J.C., Garcia, L., and Danos, O. (2004). Rescue of dystrophic muscle through U7 snRNA-mediated exon skipping. *Science* 306**,** 1796-1799.

Hibaoui, Y., Reutenauer-Patte, J., Patthey-Vuadens, O., Ruegg, U.T., and Dorchies, O.M. (2011). Melatonin improves muscle function of the dystrophic mdx^5Cv^ mouse, a model for Duchenne muscular dystrophy. *J. Pineal Res.* 51**,** 163-171.

Nakae, Y., Dorchies, O.M., Stoward, P.J., Zimmermann, B.F., Ritter, C., and Ruegg, U.T. (2012). Quantitative evaluation of the beneficial effects in the *mdx* mouse of epigallocatechin gallate, an antioxidant polyphenol from green tea. *Histochem. Cell Biol.* 137**,** 811-827.

Peeters, R.P., Wouters, P.J., Kaptein, E., Van Toor, H., Visser, T.J., and Van Den Berghe, G. (2003). Reduced activation and increased inactivation of thyroid hormone in tissues of critically ill patients. *J. Clin. Endocrinol. Metab.* 88**,** 3202-3211.

Reutenauer-Patte, J., Boittin, F.X., Patthey-Vuadens, O., Ruegg, U.T., and Dorchies, O.M. (2012). Urocortins improve dystrophic skeletal muscle structure and function through both PKA- and Epac-dependent pathways. *Am. J. Pathol.* 180**,** 749-762.

Reutenauer, J., Dorchies, O.M., Patthey-Vuadens, O., Vuagniaux, G., and Ruegg, U.T. (2008). Investigation of Debio 025, a cyclophilin inhibitor, in the dystrophic mdx mouse, a model for Duchenne muscular dystrophy. *Br. J. Pharmacol.* 155**,** 574-584.

Salvatore, D., Bartha, T., Harney, J.W., and Larsen, P.R. (1996). Molecular biological and biochemical characterization of the human type 2 selenodeiodinase. *Endocrinology* 137**,** 3308-3315.

Salvatore, D., Simonides, W.S., Dentice, M., Zavacki, A.M., and Larsen, P.R. (2014). Thyroid hormones and skeletal muscle--new insights and potential implications. *Nat. Rev. Endocrinol.* 10**,** 206-214.
